# Multimodal therapy for ovarian cancer with brain metastases diagnosed synchronously at the initial phase: case report of a long-term survivor with comprehensive literature review

**DOI:** 10.1016/j.gore.2026.102124

**Published:** 2026-05-25

**Authors:** Wissam Arab, Nadine El Kassis, Clement Khoury, Hussein Mansour, Georges Chahine, David Atallah

**Affiliations:** a Department of Gynaecologic Oncology, Hotel-Dieu de France University Hospital, Beirut, Lebanon; b Department of Radiation Oncology, Hotel-Dieu de France University Hospital, Beirut, Lebanon; c Department of Medical Oncology, Hotel-Dieu de France University Hospital, Beirut, Lebanon

**Keywords:** PARP inhibitors, Olaparib, Niraparib, Brain metastasis, Ovarian cancer, Radiation therapy, BRCA

## Abstract

Brain metastases from epithelial ovarian cancer (EOC) are uncommon and usually occur during disease recurrence. Their occurrence at the initial phase of the disease is particularly rare. Overall prognosis remains poor despite the employment of a multimodal therapy including surgery, radiotherapy and chemotherapy, with reported progression-free survivals of approximately 12 months. The role of targeted therapies in this setting, particularly poly ADP-ribose polymerase inhibitors (PARPi), remains incompletely defined.

We report the case of a 45-year-old woman with high-grade serous ovarian carcinoma who developed symptomatic brain metastases 6 days following primary debulking surgery (PDS). A BRCA1 mutation was identified on tumor sequencing and was subsequently confirmed as a germline pathogenic variant. Complete remission was obtained using stereotactic radiotherapy to the brain lesions, followed by platinum-based chemotherapy and maintenance therapy with bevacizumab and olaparib. 45 months since the initiation of olaparib, the patient still has no evidence of disease.

PARPi are a promising maintenance therapy to control EOC with secondary brain metastases, particularly in PARPi-naïve disease. Their role in this setting remains emerging and primarily supported by limited case reports and small series. Further studies are required to better define their role.

## Highlights

• Brain metastases in EOC are rare and only investigated in case of neurological symptoms.
• Brain metastasis spreads hematogenously and is linked to advanced disease, serous histology, BRCA mutation and survival.
• Treatment of brain metastases in EOC is unstandardized: SRT ± surgery plus platinum chemotherapy and bevacizumab.
• Maintenance with PARP inhibitors prolongs survival after brain metastases, especially in PARPi-naïve patients with sensitive metastatic clones.

## Graphical abstract

**Figure:**
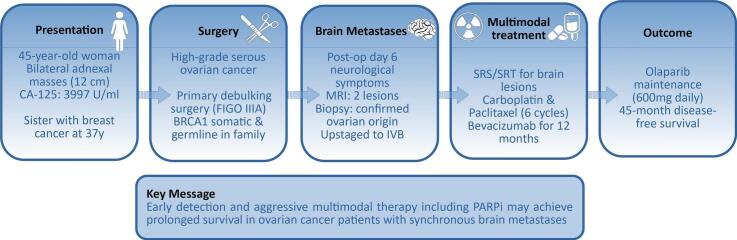
Schematic overview of the case illustrating clinical presentation, surgical management, development of brain lesions, multimodal treatment and long-term response to maintenance PARP inhibition.

## 1 Introduction

Patients with EOC frequently present with regionally disseminated or metastatic disease, most commonly affecting the peritoneum, rectosigmoid, aortic lymph nodes, spleen, pleura and liver (Cancer Staging Manual, 2017). Brain metastases arise from a variety of primary malignancies and represent a therapeutic challenge. In EOC, brain metastases constitute a rare finding, as neoplastic cells typically spread through peritoneal linings and lymphatic channels. However, their incidence appears to be increasing, likely due to improvements in overall patients’ survival and advances in diagnostic modalities (Pakneshan et al., 2014). According to a systematic review published in 2014 on 316 cases, brain metastases represent a form of extra-abdominal relapse occurring on average 2 years after the initial diagnosis of ovarian cancer. In only three cases (0.9%), brain metastases were identified at the time of initial diagnosis of EOC (Pakneshan et al., 2014).

Management of brain metastases in EOC remains challenging, and no standardised approach currently exists, given the multiple available options, including whole brain radiation therapy (WBRT), stereotactic radiosurgery (SRS), surgery and chemotherapy. Recently, with the availability of targeted therapy using poly ADP-ribose polymerase inhibitors (PARPi), prognosis of ovarian cancer with brain metastases seems to be improving. PARPi lead to an accumulation of double strand breaks particularly in patients where homologous recombination is deficient, such as those with BRCA 1–2 mutation and homologous recombination deficiency (HRD).

To our knowledge, there are very few published papers reporting the diagnosis of brain metastases during the initial phase of the disease, with only two cases receiving PARPi as part of the management strategy (Tsuchino et al., 2024 Aug, Cabitza et al., 2023 Dec 19). Hereby, we illustrate the case of a patient with BRCA-1 mutation who underwent a PDS and was diagnosed in the early postoperative period of brain metastases treated successfully with excellent progression-free survival (PFS). We also present a comprehensive review of the existing literature on the treatment of brain metastases with PARPi, allowing comparison of patient characteristics, therapeutic approaches and prognostic factors that may affect survival outcomes.

## 2 Case presentation

Our patient is a 45-year-old, para 2, heavy smoker, with a family history notable for a sister diagnosed with breast cancer at the age of 37. She presented with bloating and was found to have bilateral complex adnexal masses on ultrasonography. Pelvic MRI showed bilateral suspicious ovarian masses measuring 12 × 8 cm on the right and 12 × 6 cm on the left. Computed tomography (CT) scan of the chest, abdomen and pelvis showed no evidence of distant metastases or peritoneal carcinomatosis. CA-125 level was 3997 U/ml. A diagnostic laparoscopy certified the diagnosis of a high-grade serous EOC with a peritoneal nodule in the right iliac fossa. The patient subsequently underwent PDS, including hysterectomy with bilateral salpingo-oophorectomy, pelvic peritonectomy and ileo-cecal resection en-bloc, along with infragastric omentectomy, para-aortic and pelvic lymphadenectomy. Complete cytoreduction was achieved. Histopathological examination revealed a high-grade serous ovarian carcinoma infiltrating the meso-colon, meso-appendix and uterine serosa. Two out of 70 aortic lymph nodes returned positive, corresponding to FIGO stage IIIA (International Federation of Gynecology and Obstetrics).

**Fig. 1:**
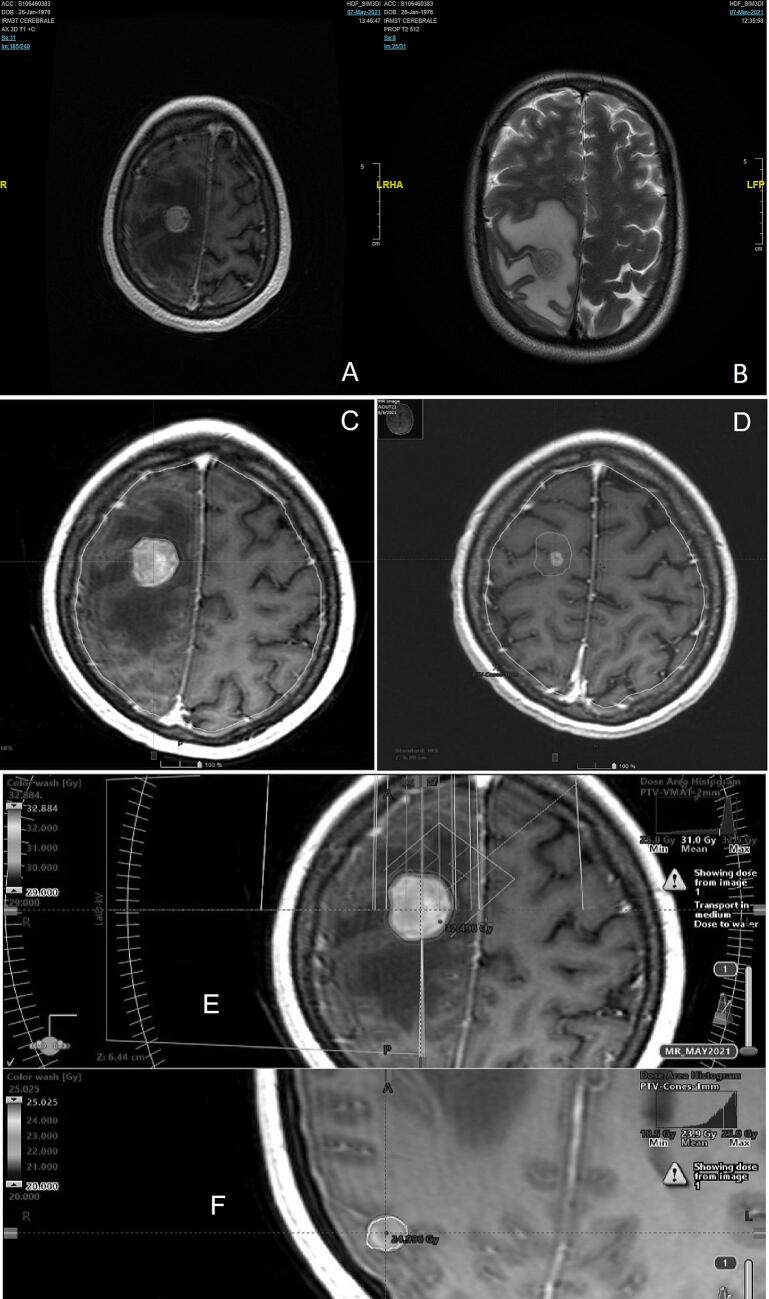
Brain MRI with gadolinium showing right parietal and fronto-parietal lesions with surrounding vasogenic edema (A&B). Treatment dosimetry in color wash representation at the right parietal metastasis (E) and the right fronto-parietal metastasis (F). Radiological response after SRT: Pre-SRT axial T1-weighted gadolinium-enhanced brain MRI showing the 20x18x21 mm right fronto-parietal (C), 10 weeks post-SRT axial T1-weighted gadolinium-enhanced brain MRI showing the extent of response at the level of the same lesion with complete resolution of the surrounding vasogenic edema (D).

**Fig. 2:**
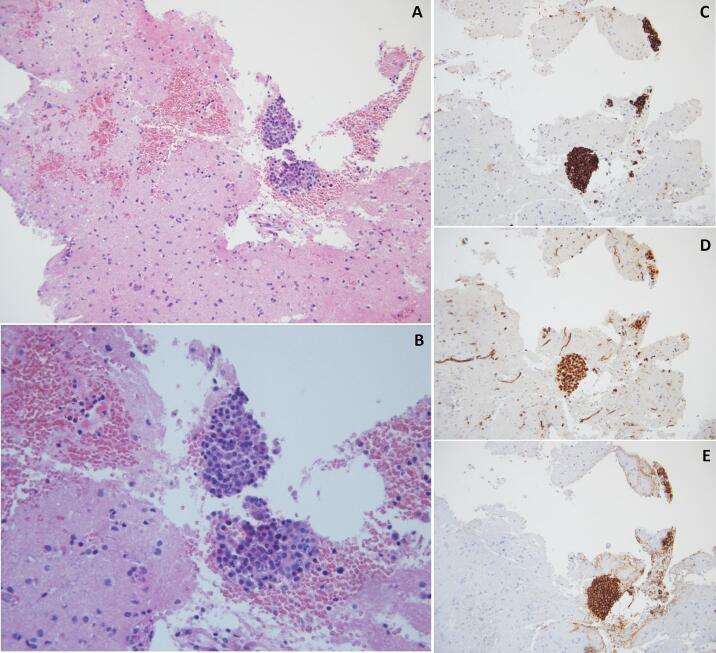
Microphotograph of the biopsied cerebral lesion: haematoxylin-eosin stain at x20 (A) and x40 magnification (B). Immunohistochemistry showing features consistent with ovarian primary origin: anti-CK7 (C), anti-WT1 (D) and anti-BerEP4 (E).

On day 6 post-PDS, the patient developed headache and focal neurological deficits including paresthesia, weakness and clonic movements of the left limbs. Brain MRI showed two ring-enhancing lesions, the largest measuring 17-mm in the right fronto-parietal lobe and the second 6-mm in the right parietal lobe, both associated with significant surrounding edema (Fig. 1A-B). A stereotactic-guided biopsy was performed and returned positive for serous high-grade carcinoma of ovarian origin (Fig. 2). The diagnosis of brain metastases led to restaging of the disease to FIGO stage IVB.

Next generation sequencing of the primary tumor identified a BRCA1 genomic alteration (p.C44F), and subsequent germline testing confirmed a pathogenic BRCA1 mutation.

The brain lesions were treated with stereotactic radiosurgery (SRS) and stereotactic radiotherapy (SRT). Treatment was delivered using a Varian TrueBEAM linear accelerator with a six-degree-of-freedom couch and iPlan treatment planning system (BrainLab). Patient positioning was stabilized with a stereotactic thermoplastic mask, allowing sub-millimetric precision. The right parietal metastasis was treated with single-fraction SRS (20 Gy) while the right fronto-parietal metastasis was treated with 3 fractions of SRT delivered one day after the other (total 27 Gy). Planning tumor volume (PTV) was generated by adding a 1-mm margin to the SRS gross tumor volume (GTV) and a 2-mm margin to the SRT GTV. Fig. 1E-F illustrate a dosimetric analysis of the treatment plans. We elected to treat our patient with SRS/SRT rather than WBRT, considering she had an excellent performance status and preserved neurocognitive function. Fig. 1C-D illustrate the radiological response 10 weeks following SRT.

After completion of local therapy, chemotherapy was initiated using carboplatin and paclitaxel for 6 cycles, in association with bevacizumab. Maintenance therapy afterwards included bevacizumab (for 12months) and olaparib, which the patient continues to receive at the time of writing of this manuscript (January 2026). Olaparib was administered at a dose of 300 mg twice daily (600 mg per day) and was well tolerated. Given the lack of robust data guiding optimal duration of PARP inhibition in this specific setting (stage IV with initial brain metastases), as well as the excellent treatment tolerance, continuation of olaparib was considered appropriate after multidisciplinary discussion. A close radiographic surveillance program was designed with brain MRI every 3 months for 2 years, and every 6 months afterwards, in addition to pelvic disease monitoring using clinical assessment every 3 months for 2 years and every 6 months afterwards, coupled with CA-125 monitoring. The patient remains disease-free after 45 months of follow-up (last follow-up visit 2 months ago).

## 3 Discussion and literature review

Screening for brain metastases in patients with EOC is not recommended in the absence of symptoms. In our case, the patient did not exhibit any neurological signs prior to surgery. The reason for the development of symptoms in the early postoperative period is unclear. One possible explanation is postoperative immune modulation, which has been suggested to facilitate the growth of existing occult metastases. This suggests that occult brain micrometastases were likely already present at the time of initial diagnosis, but remained clinically silent.

### 3.1 Risk factors and prognostic factors

The risk of brain metastases is higher in patients with advanced disease, serous histology and high-grade lesions (Pakneshan et al., 2014 Aug). Germline/somatic mutations in BRCA genes have been associated with an increased risk, but the reason behind is unclear (Ratner et al., 2019 Jun, Kasherman et al., 2021 Jan). The better survival of patients with HRD might explain the higher incidence of brain metastases as a late manifestation. In this same context, a review on EOC patients with brain metastases has shown that 71% had pathogenic BRCA mutation in the family (Jernigan et al., 2015). The case report of Favier et al. found that BRCA mutation was also detected in metastatic cells to the brain (Favier et al., 2020). Similarly, tumors with homologous recombination deficiency have demonstrated an increased ability to develop brain metastases in breast cancer models (Diossy et al., 2018). It seems that defective DNA repair in BRCA patients contributes to genetic instability, enabling tumors to acquire mechanisms for invasion and distant metastases beyond the peritoneal cavity.

Regarding prognosis, factors associated with good prognosis include single brain lesion, good performance status at diagnosis, use of a multimodal therapy and young age < 50 (Pakneshan et al., 2014). The absence of extracranial disease has also been associated with improved survival (Frezzini et al., 2024). In contrast, the number of relapses before brain involvement does not appear to affect prognosis significantly. It is also unclear whether a short interval from initial diagnosis of ovarian cancer to brain involvement could negatively affect prognosis (Pakneshan et al., 2014).

### 3.2 Treatment modalities

Treating brain metastases in EOC remains unstandardised. These patients are often excluded from clinical trials because they have previously received multiple lines of systemic therapy and may therefore exhibit resistance to several treatment agents.

In neuro-oncology, local therapy remains the cornerstone of treatment due to the heterogeneous uptake of anticancer drugs across the blood–brain barrier. WBRT is not commonly used nowadays due to the risk of sequelae and negative impact on the neurocognitive function. A combination of SRS and SRT is seen as the perfect combination. SRS/SRT is a technique that delivers single (SRS) or multiple (SRT) high-dose fractions of radiation to the brain metastases with optimal sparing of the surrounding normal brain. Prospective trials of SRS have used single doses in the range of 20–24 Gy for brain metastases less than 2 cm (Yamamoto et al., 2014). However, metastases ≥ 2 cm had to be treated with lower single doses (15–18 Gy) to mitigate the risk of radiation necrosis (Milano et al., 2021). A study comparing SRS (15–18 Gy) versus SRT (27 Gy in 3 fractions) demonstrated that multifraction treatment was associated with higher local control and lower rates of necrosis (Milano et al., 2021).

Surgery has also been considered for local control of brain metastases in EOC: in selected patients with surgically accessible solitary lesions, surgery followed by local radiation has yielded excellent outcomes (Frezzini et al., 2024).

The addition of systemic therapy to local treatment has been associated with improved outcomes and more durable systemic disease control. Chemotherapy using platinum agents is particularly beneficial in this context, as platinum agents are believed to penetrate the blood brain barrier (BBB) when administered following radiotherapy. Consistent with this, our patient achieved a complete response following SRS/SRT and subsequent chemotherapy.

PARP inhibitors have demonstrated significant benefit as maintenance therapy in ovarian cancer following PDS and have also been approved for the treatment of platinum-sensitive recurrent disease. The SOLO-1 and PAOLA-1 trials showed significant improvements in PFS with these agents in patients with BRCA mutations and homologous recombination deficiency (Ray-Coquard et al., 2019 Dec 19, Moore et al., 2018 Dec 27). Furthermore, preclinical studies suggest that PARP inhibitors can penetrate the blood–brain barrier, raising the chance of their contribution in intracranial disease control (Tentori et al., 2005).

### 3.3 Literature review

A literature search was conducted using PubMed and Embase databases to identify reports of EOC with brain metastases in which PARP inhibitors were used as part of the treatment strategy. The search included combinations of the terms “ovarian cancer”, “brain metastases”, and “PARP inhibitor” or “olaparib” or “niraparib”. Only English-language case reports and case series with available clinical outcome data were included. The search identified 15 case reports and 3 case-series, representing a total of 60 reported cases.

Mohler et al. reported a case-series of 5 patients diagnosed with brain metastases at recurrence. Three of five were BRCA positive, and all patients received olaparib following brain involvement. Mean survival following CNS recurrence was 22.8 ± 12 months (Mohler et al., 2021).

Sliwinska et al. published a case-series of 5 patients that were also diagnosed with brain metastases at recurrence. Brain metastases were identified in 2 patients prior to PARPi therapy, and in 3 patients after its completion. 3/5 were BRCA-positive, and the median survival following brain involvement was 8 months (Sliwinska et al., 2023).

A national multicentre case-series from the United Kingdom reported 35 patients treated for brain metastases in ovarian cancer. All the patients had brain involvement upon relapse. 15 patients did not receive PARPi until brain metastasis, while 14 patients had PARPi before brain metastasis, which occurred as a second or third relapse. Among PARPi-naïve patients, 13 of 15 had brain metastases as their first relapse, and their median PFS was 20 months. In contrast, patients previously treated with PARPi experienced inferior outcomes with a median PFS of only 7 months following diagnosis of brain metastases (Alizzi et al., 2023).

In addition to these case-series, 15 individual case reports described the use of PARPi in the management of brain metastases from EOC. Table 1 summarises their related clinico-pathological features, treatment and outcome data. Outcomes show that PARPi can prolong survival and delay relapses, with a median PFS of 20 months following diagnosis of CNS disease (Tsuchino et al., 2024 Aug, Cabitza et al., 2023 Dec 19, Kasherman et al., 2021 Jan, Favier et al., 2020 Apr 27, Frezzini et al., 2024 Jul 18, Alizzi et al., 2023 Mar 25, Bangham et al., 2016 Nov, Gallego et al., 2021 Sep, Gray et al., 2019 Aug, Sakamoto et al., 2019 Mar). Of the 15 cases, two had brain involvement at the initial stage and received olaparib and niraparib, achieving a PFS of 25 and 27 months, respectively. The remaining 13 cases developed brain metastases at recurrence, and in 11 of these 13 cases, PARPi were introduced only after brain involvement. Of the 15 cases listed in table 1, only four had a PFS below 12 months following brain involvement. Of these four cases, two were already exposed to PARPi either initially or at first relapse, therefore prior to CNS involvement. These findings meet those reported by the case-series published in the UK (Alizzi et al., 2023). Taken together, these observations suggest that prior exposure to PARP inhibitors may influence subsequent treatment response, potentially reflecting the emergence of PARP inhibitor resistance mechanisms. In this context, disease may behave similarly to the well-described paradigm of platinum-sensitive versus platinum-resistant ovarian cancer.

**Table 1:** Clinico-pathological features, treatments and survival outcomes of patients with brain metastases from EOC

Authors	Age	Stage at diagnosis	BRCA or HRD	Timing (number) of BM	Extracranial lesions at diagnosis of BM	Timing of relapses (months)	Local therapy for BM	Systemic therapy (chemotherapy/targeted therapy)	PFS following BM (months)	OS since initial phase (months)
Tsuchino (2024)	74	IVB	Germline BRCA-2	Initial (multiple)	Lungs, carcinomatosis	24 45	SRT	TC (initially) TC + Olaparib (platinum sensitive relapse at 24 m)	25	57
Tsuchino (2024)	39	IIIC	None	Relapse (multiple)	Lymph nodes	24	SRT	TC (initially) TC + Olaparib (platinum sensitive relapse at 24mo)	25	63
Frezzini (2024)	53	IIIC	Germline BRCA-1	Relapse (single)	Lymph nodes	25	Surgery SRS	TC + Bev (initially) CPLD + Niraparib (platinum-sensitive relapse at 25mo)	32	Alive at writing
Frezzini (2024)	52	IIIC	Germline BRCA-1	Relapse (single)	None	49	SRS	TC – BEV (initially) C (platinum sensitive relapse at 49mo)	63	Alive at writing
Frezzini (2024)	47	IIIB	Germline BRCA-1	Second relapse (multiple)	None	68 118	SRS	TC (initially) CPLD (relapse at 68mo) TC and Olaparib (relapse at 118mo)	21	150
Frezzini (2024)	65	IIIC	None	Second relapse (single)	None	35 49	Surgery SRS	TC (initially) TC and Niraparib (35mo) Niraparib (49mo)	12	61
Frezzini (2024)	52	IIB	Germline BRCA-1	Second relapse (single)	None	20	Surgery SRS	TC (initially) CPLD Olaparib (20mo)	18	Alive at writing
Frezzini (2024)	73	IIIA	None	Relapse (single)	None	20	Surgery SRS	TC Niraparib (initially) Niraparib (relapse 20mo)	10	Alive at writing
Cabitza (2023)	<50	IVB	None	Initial (single)	Pelvis	−	Surgery	TC + Niraparib (initially)	27	Alive at writing
Kasherman (2021)	47	IIIC	Germline BRCA-1	Relapse (multiple)	None	12	SRS	TC (initially) CG + Olaparib (relapse at 12mo)	11	Alive at writing
Gallego (2021)	51	IIIC	Germline BRCA-1	Relapse (multiple)	Mediastinal lymph nodes	17	WBRT	TC (initially) CG + Olaparib (relapse at 17mo)	42	Alive at writing
Gray (2020)	68	IIIC	Germline BRCA-1	Relapse (multiple)	None	19	WBRT	C (initially) CG + Niraparib (relapse at 19mo)	17	Alive at writing
Favier (2020)	54	IIIC	Somatic BRCA-2	Second relapse (multiple)	None	24 30	WBRT	TC (initially) Oxaliplatin (first relapse at 24mo) C + Olaparib (second relapse at 30mo)	14	16
Sakamoto (2019)	58	IIIC	Germline BRCA-1	Relapse (multiple)	None	11	WBRT	TC (initially) TC + Olaparib (relapse at 11mo)	22	Alive at writing
Bangham (2016)	61	IVB	Germline BRCA-2	Relapse (single)	None	24	Surgery SRS	TC (initially) C + Olaparib (relapse at 24mo)	12	Alive at writing

Abbreviations: BM: Brain Metastases, TC: Paclitaxel Carboplatin, CPLD: carboplatin pegylated liposomal doxorubicin, Bev: Bevacizumab, CG Carboplatin Gemcitabin, mo: months.

In our case, several good prognostic factors were present, including young age, good performance status, absence of extracranial metastases, use of multimodal therapy, achievement of complete cytoreduction and occurrence of brain metastases before administration of any treatment. Our case illustrates one of the longest progression-free survivals reported following early brain metastasis in epithelial ovarian cancer. The other two cases reported PFS of 25 and 27 months, despite surgical excision in one case (Cabitza et al., 2023 Dec 19, Tsuchino et al., 2024 Aug). In their report, Tsuchino et al. described a 74-year-old patient presenting with advanced epithelial ovarian cancer associated with a BRCA2 mutation and synchronous metastases involving the brain, lungs, and abdominopelvic cavity. The authors also included a literature review summarizing nine reported cases of brain metastases in epithelial ovarian cancer managed with PARP inhibitors. In contrast, our study expands the literature review by including a larger number of reported cases, and provides a structured analysis of treatment strategies and outcomes. Furthermore, our case differs in several clinically relevant aspects from the case reported by Tsuchino et al. Our patient harbored a germline BRCA1 mutation, thereby adding biological diversity to the limited available data. In addition, our case demonstrates prolonged progression-free survival of 45 months, which exceeds most outcomes reported to date. Taken together, our findings contribute additional evidence supporting the potential role of PARP inhibitors as part of a multimodal treatment approach in selected patients with ovarian cancer and brain metastases.

Several questions remain unresolved, particularly concerning the optimal management of patients who experience progression of brain metastases on PARPi and in the absence of extracranial disease. One potential strategy may involve switching to niraparib, which has demonstrated greater blood–brain barrier penetration and stronger tumor inhibition in preclinical models (Mikule and Wilcoxen, 2015). Clinical reports have also described favorable responses to niraparib in platinum-sensitive intracranial relapse, including in patients with BRCA wild-type tumors (Cabitza et al., 2023 Dec 19, Gray et al., 2019 Aug). There are clear benefits in continuing PARPi for oligometastatic relapse, the rationale being maintenance of systemic control over a presumably stable disease under PARPi, with use of local therapy to PARPi-resistant clones (Frezzini et al., 2024).

Finally, we agree with other authors who stressed on the importance of sharing these cases in the literature. Creating an international registry in order to improve the management of brain metastases from EOC is necessary. Also, enrolling patients with brain metastases in prospective trials testing PARPi effectiveness should be encouraged.

In conclusion, brain metastases at the time of initial management of EOC remain extremely rare. Our case suggests that aggressive multimodal management combining stereotactic radiation, platinum-based chemotherapy and PARP inhibition may lead to durable disease control in selected patients with BRCA-mutated disease.

## 4 Ethics statement.

The authors confirm that written consent for submission and publication of this case report, including the images and associated text, has been obtained from the patient.

The ethics committee of Saint Joseph University – School of Medicine waived its’ approval for this case report.

## 5 Authors’ contribution

Wissam Arab: Study conception, literature review, manuscript drafting, and critical review of the manuscript.

Nadine El Kassis: Literature review and drafting of the discussion.

Clement Khoury: Provided radiation therapy details and drafted case presentation.

Hussein Mansour: Drafted the introduction and participated in literature review.

Georges Chahine: Literature review regarding targeted therapy and drafting of the discussion.

David Atallah: Study conception, overview of the work and critical revision of the discussion.

## 6 Data availability

All data regarding the patient’s treatment are available upon request.

## CRediT authorship contribution statement

**Wissam Arab:** Writing – review & editing, Writing – original draft, Visualization, Supervision, Methodology, Data curation. **Nadine El Kassis:** Supervision, Software, Resources, Methodology, Investigation. **Clement Khoury:** Writing – original draft, Visualization, Software, Resources. **Hussein Mansour:** Resources, Methodology, Investigation. **Georges Chahine:** Visualization, Validation, Supervision. **David Atallah:** Writing – review & editing, Validation, Resources, Project administration, Data curation, Conceptualization.

## Funding

There has been no significant financial support for this work that could have influenced its outcome.

## Declaration of Competing Interest

The authors declare that they have no known competing financial interests or personal relationships that could have appeared to influence the work reported in this paper.
